# Editorial: Philosophical perspectives on qualitative psychological and social science research

**DOI:** 10.3389/fpsyg.2023.1237980

**Published:** 2023-07-17

**Authors:** Gillie Gabay, Paul M. W. Hackett, Christopher Hayre

**Affiliations:** ^1^Achva Academic College, Arugot, Israel; ^2^University of Wales Trinity Saint David, Carmarthen, United Kingdom; ^3^School of Communication, University of Suffolk, Ipswich, United Kingdom; ^4^Nnamdi Azikiwe University, Awka, Nigeria; ^5^University of Exeter, Exeter, United Kingdom

**Keywords:** bricolage, declarative mapping sentence, life stories, facet theory, methodology, philosophy, theory, psychology

We have written this editorial commentary as an endpoint to what we consider to be a successful Research Topic: “*Philosophical perspectives on qualitative psychological and social science research.”* Our first impetus in proposing the topic was to examine a plethora of perspectives of research within psychology and social sciences. Issues associated with philosophical perspectives on qualitative psychological and social science research related to the nature of the data, its collection, the types of inquiries, the form of questions, the rigor and other issues associated with information analysis, the positionality of the researcher, and the processes required to yield meaningful, trustworthy results, as well as reflexivity. Our second guiding intention was to adopt a broad philosophical approach, one of many that a researcher may consider, to allow for the exploration of the nature of the research process itself and how this process influences the development of knowledge from research findings across a broad range of approaches and methodologies.

A key feature of this Research Topic is how various methodologies contribute to the current knowledge and inform the literature of psychology and social science research. The authors of this Research Topic have presented fascinating, diverse, theoretical concepts, methodologies, and philosophies, carrying similarities and differences among them as expressions of their research philosophy.

## Highlights from the article collection

In this editorial, we do not simply want to repeat the abstracts that can be found for each contribution; rather, we have provided a brief reflection of some of the numerous highlights that are present in the articles included in this Research Topic. We first wish to mention the breadth of perspectives of the articles included in the Research Topic, which is demonstrated in many ways. For example, articles on this topic embody many different ways to approach qualitative social science research, namely, facet theory,^1^ bricolage,^2^ life stories, and psychological phenomenology.^3^ Furthermore, the inquiries are of varying designs such as cross-sectional, longitudinal, intervention studies, and a review. These articles highlight individual, communal, organizational, and contextual factors that affect the daily lives of children and adults from Canada, Chile, England, Israel, Germany, and South China. The highlights of the seven articles provide a sense of their themes and novel contributions.

Gordley-Smith reviews Hackett's book on the fundamentals of facet theory and mapping sentences. Hackett expanded facet theory from quantitative methods into qualitative approaches. The mapping sentences^4^ reviewed the time period from the field of avian cognitive behavior (Hackett, 2019) and prison officers' occupational stress (Small and Hackett, 2023) to the Black Lives Matter movement (Hackett and Schwarzenbach, 2020). Furthermore, adopting facet theory and the mapping sentence, Rabenu and Shkoler opened a hatch to their ongoing research into the area of consumer psychology research and the marketing of higher education. The authors delved into concepts under investigation and defined them systematically, comprehensively, and parsimoniously.

López-Montero et al. followed the trend that supports the term “life story” instead of “life history” to investigate human experiences. Li and Liu  also investigated human experiences by comparing structures using psychological phenomenology within pedagogic neoliberalism.

Ben-Asher presented the virtue of the Bricolage (see text footnote 2), following the recommendation to regard the bricolage methodology as an analytical alternative to methodological templates. This approach has the potential to improve the way researchers understand, formulate, and implement methodological choices in their research (Pratt et al., 2022).

Martínez-Pernía et al. studied the empathic experience of pain using an experimental phenomenological method by analyzing videos of sportspersons suffering physical accidents while practicing extreme sports.

They demonstrated how phenomenological data (see text footnote 3) may contribute to comprehending empathy for pain in social neuroscience. The authors also addressed the phenomenological aspect of the enactive approach to the three dimensions of an embodiment of human consciousness, especially the intersubjective dimension. Finally, Fisher and Refael Fanyo's study (quantitative) examined how the level of communal affiliation affects the perceived authority of elementary school teachers and the involvement of parents.

After briefly considering what we feel are the highlights of the Research Topic, we have in the following section commented on how we believe this Research Topic contributes to the field of research.

## Contribution of the Research Topic to the field of research

Articles in this Research Topic are from researchers from around the globe. The inclusion of culturally diverse participants and researchers enriches psychology and social science research. The different perspectives of the researchers and their philosophies provide new methodological insights for the sake of both scientific knowledge and for applied purposes.

As for specific contributions, the two articles on facet theory illustrated that declarative mapping sentences may stimulate cumulative knowledge development and create space for knowledge emergence. Further, Hackett demonstrated how Facet theory and the mapping sentence offer a potentially constructive philosophical basis for understanding daily existence. Rabenu and Shkoler demonstrated how facet theory can be used to define new concepts in a systematic, comprehensive, and yet parsimonious manner.

Since life stories in qualitative methods were thus far studied using a multiplicity of techniques, lacking a clear delimitation, López-Montero et al. unraveled the possible confusion that may be present due to this delimitation and reviewed techniques to study human experiences and innovatively apply bibliometrics to show how the centrality of knowledge transferred from psychology (Neumann, 2015). In addition, studying life stories, Li and Liu used phenomenological psychology (see text footnote 3) to reach the typical essence based on structures derived from each case that can be compared with results from similar studies_._

Ben-Asher article on Bricolage, which involves combining resources for a new purpose, illustrated how practices were creatively designed to fit the research, acknowledging that there are many possible ways of conducting high-quality qualitative research.

Martínez-Pernía et al. extended the social cognition research which has been traditionally conducted through self-report, behavioral, and neuroimaging measures to first-person methods based on the phenomenological experience (Giorgi and Palmisano, 2017).

Finally, Fisher and Rafael Fayol's study carries practice implications for formulating policies to improve the school-parent relationship and strengthen the teacher's authority.

## Concluding thoughts and looking forward

The original goal set by the editors of this Research Topic was to foster an interdisciplinary exchange of some of the research methodologies and philosophies that are employed by researchers to comprehend various phenomena, events, and states of affairs to allow the creation of new knowledge. The breadth of the studies in this Research Topic, including studies by researchers from the fields of education, marketing, consumer psychology, neuroscience, social cognition, and psychology, exceeded our expectations.

The variety of perspectives and the use of diverse methodologies and philosophies are vital to working across fields in the thriving, rich field of psychology and social sciences research. Future studies may continue to develop additional ways to conduct their research using various techniques from within different disciplines and by applying different methodologies. Furthermore, continued international collaborations can establish new methodologies which may lead to further development in these disciplines, based on multidisciplinary/cross-disciplinary and multi-cultural/cross-cultural evidence. Thus, this Research Topic presents contemporary scholarship which provides evidence that illustrates what can be learned from a plethora of studies focusing on the philosophical bases and methods employed in research conducted in psychology and the social sciences.

## Author contributions

GG wrote the initial draft of this editorial. PH made a significant contribution to the editorial. CH edited and contributed to the editorial. All authors contributed to the article and approved the submitted version.

## Footnotes

^1^Facet theory is a multi-dimensional research approach from within social science research (Canter, 1985; Hackett, 2021). Facet theory was originally a purely quantitative methodology that considered the totality of the research project from research design, data collection and analysis, and report writing. More recently, it has been extended into the realm of qualitative research.

^2^Bricolage is a qualitative critical research approach that considers research phenomena from multiple theoretical points of view. It is a multi-perspectival, multi-theoretical, and multi-methodological approach that has its roots in practices that are eclectic and employ an emergent design. Moreover, bricolage is characterized by its flexibility and plurality.

^3^Psychological phenomenology is a psychological research approach that employs phenomenological, hermeneutic, and existential philosophies in its attempts to elucidate the structures of meaning and the lived experience of research participants in a sensical manner.

^4^Mapping sentences are the major components of facet theory. A mapping sentence is a sentence written in ordinary prose that contains a clear specification of a research project, including participants, content variables and/or areas of research interest, and pertinent background characteristics. In quantitative research, the mapping sentence also contains a specification of the outcome measures, but this is not present in qualitative research that uses a declarative mapping sentence (Hackett, 2020; Hackett and Li, 2022).
